# Evaluating the utility of ST elevation in lead II > lead III in differentiating pericardial disease from STEMI

**DOI:** 10.1186/1757-7241-20-S2-P20

**Published:** 2012-04-16

**Authors:** Daniel Henning, Cecilie Markvard Moeller, Alexander Fjaeldstad, Michael Fogel, Christopher Fischer, Edward Ullman

**Affiliations:** 1 Dept. of Emergency Medicine, Beth Israel Deaconess Medical Center (BIDMC), Boston, USA; 2 Research Center for Emergency Medicine, Aarhus University Hospital, Denmark

## Background

Accurate diagnosis of ST elevation myocardial infarction (STEMI) is complicated by the presence of mimickers such as pericarditis, one of the most common reasons for (negative) emergency cardiac catheterization. Beyond common electrocardiogram (ECG) criteria for pericarditis, a rule of ST segment elevation in lead II greater than lead III (II > III), has been described in literatures and lectures to suggest pericardial disease (PD) and not STEMI.

The objective of this study is to define the operating characteristics for the ability of the II > III rule to discriminate PD from STEMI.

## Methods

A retrospective cohort study of all patients from an academic emergency department (ED) with the diagnosis of PD (pericarditis, pericardial effusion, pericardial tamponade) or inferior STEMI from 2005-2009 was performed. Inferior STEMI patients were selected as ST elevation in leads II and III. Patients without an ECG in the ED were excluded. Diagnoses were defined by final ECG interpretation, echocardiogram, and by cardiac catheterization. The rule was defined as positive if lead II > III (PD+). The first ED ECG for each patient was randomized and presented without a clinical history to an ED attending physician to apply the rule. A second physician was asked to apply the rule to determine reproducibility. We calculated a kappa score for agreement along with the operating characteristics of the rule.

## Results

We enrolled 283 patients: 122 with PD and 161 with inferior STEMI. When the rule was PD+, indicating PD and not STEMI, the positive predictive value was 19/32 (59%); whereas, if the rule was absent, the negative predictive value was 148/251 (59%). Or, among those with PD, sensitivity was 19/122 (16%, 95% confidence intervals; 10-23%) and specificity was 148/161 (92%, 95% confidence intervals 87-95%). There was moderate but significant agreement (kappa=0.65, p<0.01) between ECG readers.

## Conclusion

Despite suggestions that II > III aids the ECG diagnosis of PD, this study suggest that the II > III rule does not have a level of diagnostic accuracy reliable for clinical decision-making. This study is limited by the fact that we artificially altered population prevalence, by only including PD and STEMI ECGs.

